# Humor Styles Are Related to Loneliness Across 15 Countries

**DOI:** 10.5964/ejop.5407

**Published:** 2022-11-30

**Authors:** Julie Aitken Schermer, Radosław Rogoza, Marija Branković, Oscar Oviedo-Trespalacios, Tatiana Volkodav, Truong Thi Khanh Ha, Maria Magdalena Kwiatkowska, Eva Papazova, Joonha Park, Christopher Marcin Kowalski, Marta Doroszuk, Dzintra Iliško, Sadia Malik, Samuel Lins, Ginés Navarro-Carrillo, Jorge Torres-Marín, Anna Wlodarczyk, Sibele D. Aquino, Georg Krammer

**Affiliations:** 1 Management and Organizational Studies, Department of Psychology, Faculty of Social Science, University of Western Ontario, London, ON, Canada; 2 Institute of Psychology, Cardinal Stefan Wyszyński University, Warsaw, Poland; 3 Department of Psychology, Faculty of Media and Communications, Singidunum University, Belgrade, Serbia; 4 Delft University of Technology, Faculty of Technology, Policy and Management, Section of Safety and Security Science, Delft, The Netherlands; 5 Department of Pedagogy and Psychology, Kuban State University, Krasnodar, Russian Federation; 6 Faculty of Psychology, University of Social Sciences and Humanities, Vietnam National University, Hanoi, Vietnam; 7 Institute for Research in Education, Sofia, Bulgaria; 8 School of Management, NUCB Business School, Nagoya, Japan; 9 Centre for Social Cognitive Studies, Jagiellonian University, Krakow, Poland; 10 Institute of Humanities and Social Sciences, Center of Sustainable Education, University of Daugavpils, Daugavpils, Latvia; 11 Department of Psychology, University of Sargodha, Sargodha, Pakistan; 12 Laboratory of Social Psychology, Center for Psychology, University of Porto, Porto, Portugal; 13 Department of Psychology, University of Jaén, Jaén, Spain; 14 Department of Research Methods in Behavioral Sciences, University of Granada, Granada, Spain; 15 Escuela de Psicología, Universidad Católica del Norte, Antofagasta, Chile; 16 Department of Psychology, Pontificia Universidade Catolica do Rio de Janeiro, Rio de Janeiro, Brazil; 17 Institute for Educational Sciences, University College of Teacher Education Styria, Graz, Austria; Maria Grzegorzewska University, Warsaw, Poland

**Keywords:** humor styles, gender, human, adult, loneliness

## Abstract

The relationships between self-report loneliness and the four humor styles of affiliative, aggressive, self-defeating, and self-enhancing were investigated in 15 countries (N = 4,701). Because loneliness has been suggested to be both commonly experienced and detrimental, we examine if there are similar patterns between humor styles, gender, and age with loneliness in samples of individuals from diverse backgrounds. Across the country samples, affiliative and self-enhancing humor styles negatively correlated with loneliness, self-defeating was positively correlated, and the aggressive humor style was not significantly related. In predicting loneliness, 40.5% of the variance could be accounted. Younger females with lower affiliative, lower self-enhancing, and higher self-defeating humor style scores had higher loneliness scores. The results suggest that although national mean differences may be present, the pattern of relationships between humor styles and loneliness is consistent across these diverse samples, providing some suggestions for mental health promotion among lonely individuals.

Loneliness is a social problem experienced by many individuals. People have an essential human need to belong to a community and maintain social bonds (Baumeister & Leary, 1995; Lavigne et al., 2011). When an individual has no group to belong to, he/she experiences a sense of loneliness (Rokach, 2018). Individuals adjust to the demands of social interactions in a variety of patterns. These patterns might be more or less adaptive (Eder, 1990). In their social interactions, some people use humor in a way that facilitates positive social interaction, while others may use humor in a less prosocial manner (Martin, 2007). As loneliness may be partially explained by the use of specific humor styles in interpersonal situations, this study investigates the relationships between self-report loneliness and four humor styles in 15 countries (*N* = 4,701).

## Loneliness

Loneliness is a complex and multidimensional phenomenon, involving the feeling of absence of attachments, relations, and social bonds, resulting in sadness and frustration (Maes et al., 2016). Loneliness negatively affects one’s physical and psychosocial life and can increase the likelihood of symptoms of depression, social anxiety, poor physical health, and even cognitive decline (Cacioppo et al., 2015; Hawkley & Cacioppo, 2010; Ilhan, 2012; Tharayil, 2012; Ursin & Eriksen, 2004; Weiss, 1973). Loneliness has become a feature of contemporary society and some researchers point to an existential loneliness as an element of the human condition linking it to the fundamental ontological condition (Ettema et al., 2010). MacDonald and associates (2020a) recently found that those who report higher loneliness also score higher on a new measure of anti-mattering, suggesting that those who feel lonely also do not feel that they matter to others. In addition, loneliness has been found to be more than a situation variable or a transitory mood or emotion. In recent behavior genetic studies, loneliness has been reported to have a genetic component, with heritability estimates ranging from 35% in a large twin sample from Australia (Schermer & Martin, 2019) to 37% in a large sample from the Netherlands (Distel et al., 2010). These findings suggest that loneliness is an important individual difference variable, requiring further understanding.

In a recent report, Barreto et al. (2021) investigated loneliness from over 46,000 individuals from “273 countries, islands, and territories” using the data from the BBC Loneliness Experiment. Analyzing the item of how frequently individuals experienced loneliness, Barreto et al. (2021) reported that loneliness decreased with age, was higher in individualistic countries, and that men scored higher than women. These results suggest that loneliness may vary across samples based on where the sample was taken geographically and may vary due to the composition of the sample with respect to age and gender.

With respect to age, it is of interest that Barreto et al. (2021) reported that older people were less lonely than were younger people in their variable of frequency of experiencing loneliness. Other research has indicated that older adults are more vulnerable to social loneliness, which is partially explained by their reduced social activity, living alone or widowhood (Schiau, 2016). Recently, MacDonald et al. (2020b) demonstrated in a large sample of Dutch adults, that loneliness had a curvilinear relationship with age such that both the younger and the older were the loneliest. How age is related to loneliness and types of loneliness is an area which has not been definitely explained.

Gender has also been examined with respect to loneliness. Similar to the findings with age, the pattern of results is quite inconclusive. For example, Mullins et al. (1996) report a higher level of loneliness among senior men compared to women in the USA. By contrast, women tend to report that they experience loneliness more in some studies (Victor & Yang, 2012), whereas a meta analyses of 30 loneliness studies indicated that men are typically lonelier (Rokach, 2018). In Scandinavian countries, the level of loneliness is higher among women due to their longer lifespan and experience of widowhood and many losses (Jylhä, 2004). And, as described above, Barreto et al. (2021) reported that men had a higher frequency of loneliness. Because gender differences in loneliness may be age related or geographically related, in addition to other interpersonal variables such as personality and life circumstances, gender differences will be explored in the present study by examining the point-biserial correlations between gender and loneliness for each country sample as well as for the complete sample.

Studies have demonstrated differences in the level of loneliness across cultures. Often culture classification is on the degree of individualism versus collectivism, as was done in the study by Barreto et al. (2021). In more individualistic cultures, psychological autonomy is valued quite highly and may lead to social alienation and loneliness. In collectivist cultures, greater social support may lead to a greater sense of interpersonal connectedness (Maes et al., 2016). For example, in Western Europe, with prevailing individualism and weaker community ties, the level of loneliness is higher when compared to Eastern European cultures (Rokach, 2018), while Northern European cultures show high levels of isolation and loneliness (Dykstra, 2009). The dimension of individualism has been suggested to be a distinct predictor of loneliness in cross-culture research (Oyserman et al., 2002). However, one study of loneliness carried out in 25 countries revealed that the level of loneliness is much higher in Eastern Europe as compared with Western European and Northern European countries, with the highest level of loneliness reported in post-communist countries, such as Ukraine, Latvia, Russia, Hungary, Poland Romania, and Bulgaria (Yang & Victor, 2011). This finding might be explained by the political and economic transformations and hardships in these countries, suggesting that there may be other relevant dimensions of cultural differences beyond individualism and collectivism in influencing loneliness. The present study adds to this body of research by examining self-report loneliness from 15 diverse geographical samples.

## Humor and Humor Styles

Humor is one of the first social indicators (after crying) transmitted to babies. As early as about four months of age, children begin to smile and laugh in response to the actions of adults (Martin, 2007). The idea that the mechanism of humor exists as part of the human nature is evidenced by the fact that children who are born deaf and blind can laugh without these senses (Martin, 2007).

Martin et al. (2003) developed a comprehensive understanding of humor, based on a theoretical framework that covers the day–to–day functions of humor—adapting and dealing with crises, creating and maintaining close interpersonal relationships, bringing people together in a group, and established a research method to operationalize these significant functions. In this model, humor is multidimensional, encompassing four styles of humor. These styles are based on in-depth analyses of theoretical literature to determine what forms, functions, and styles (manners, types) of humor are considered adaptive or maladaptive in our relationships with others. Martin et al. (2003) emphasize that positive and negative manifestations, as well as the target of the humor, are opposite and relatively independent of one another, forming four dimensions or styles of humor. The affiliative humor style describes people who tend to tell funny stories, witty teases, and entertain others (Martin et al., 2003). In this way, they successfully reduce interpersonal tension and strengthen their relationships with others (Lefcourt, 2001). The self-enhancing humor style reflects a general humorous outlook and a tendency to be entertained by the inconsistencies and absurdities of everyday life (Martin et al., 2003). People who apply this style to their daily routine maintain a humorous perspective when facing stress and distress (Kuiper, Martin, & Olinger, 1993). High scores on the aggressive humor style refer to the use of sarcasm, ridicule, humiliation, offending, or ignoring others (Martin et al., 2003). This style also involves the use of humor to manipulate others by mocking or threatening (Janes & Olson, 2000). The fourth humor style, self-defeating, involves the overuse of negative jokey comments that may affect the self-concept. Engaging in this style aims at entertaining others even if it is accompanied by emotional discomfort for them. Thus, a person who engages in this style allows himself or herself to be the center of their mockery and sarcastic remarks (Martin et al., 2003). At the same time, the self-defeating humor style is a defense mechanism by which the individual suppresses or pushes aside negative experiences, concealing emotional states and avoiding dealing with specific intrapsychic problems (Kubie, 1971). Schermer et al. (2015) reported that there were significant positive genetic correlations between the self-defeating humor style and borderline personality disorder dimensions (in a non-clinical sample), including affect instability, identity disturbance, negative relationships, and of great concern, self-harm, suggesting that self-defeating humor positively relates with loneliness possibly due to the negative relationships indicative with borderline personality disorder dimensions.

## Humor and Loneliness

Some studies have focused on humor to deal with loneliness. Humor is one possible coping mechanism with loneliness, life hardships, and changes in one’s life. As reviewed above, humor may involve a constructive outlook on life, which assists with coping with difficulties or loss. Humor may also facilitate building healthy relationships and bonds.

With respect to the four humor styles, Martin et al. (2003) suggest that individuals may use humor to deal with loneliness. Hampes (2006) reported that higher loneliness scores were negatively related to affiliative and self-enhancing humor styles but positively related to the self-defeating humor style. The same pattern of relations was revealed by a study done in Turkey (Çeçen, 2007). Zhao et al. (2012) studied how humor styles and self-esteem mediated the relation between shyness and loneliness in the Asian culture. They reported a positive correlation between the self-enhancing humor style and self-esteem. Further, they showed that shy people become lonely in part due to not displaying self-enhancing and affiliative humor. The authors stressed the importance of both of these styles of humor for adaptive social functioning in the context of a collectivist culture. In addition, male students who tend to use less of the self-enhancing humor style were at a higher risk of loneliness compared with female students (Zhao et al., 2012). In comparing Hong Kong and mainland China, Yue et al. (2014) reported that self-enhancing and affiliative styles of humor predicted lower loneliness scores. The self-defeating humor style emerged as a significant predictor of social and emotional loneliness among Hong Kong students, but only for social loneliness among the mainland Chinese.

Fitts et al. (2009) also found that people who engage in affiliative and self-enhancing humor styles are less lonely while people who use the self-defeating style are more prone to loneliness. This study further showed how humor styles mediated the link between shyness and loneliness through the finding that shy people use humor styles detrimental to their self and fail to use socially adaptive humor styles. Further, Schermer et al. (2017) explored genetic aspects in the study of humor styles and loneliness and reported phenotypic, genetic, and environmental correlations between the humor style scales and a loneliness scale. This study revealed that, at the phenotypic level, both adaptive humor styles (affiliative and self-enhancing) are negatively related with loneliness, while the two maladaptive dimensions (aggressive and self-defeating) are positively related to loneliness. In addition, the study suggested that these phenotypic correlations are attributable to shared familial and unique environmental factors.

## Hypotheses

Accumulated evidence supports relationships between humor styles and loneliness. We hypothesized that affiliative humor styles and self-enhancing humor-styles are negatively related with loneliness, whereas the self-defeating humor style is positively related with loneliness. Although the results described above were mixed with respect to the aggressive humor style, we predicted that the aggressive humor style would have a non-significant correlation with loneliness. Our inspection of the literature led us to expect that general patterns for the relationships would be culturally invariant. However, in that cultures have different emphases on human values, it is possible that the degree of relationships between different humor styles and loneliness may differ across the country samples. Therefore, the current study aimed to illuminate geographical differences and similarities in use of different humor styles and their relationships with loneliness.

## Method

### Participants and Procedure

The sample consisted of 4701 participants (3201 females (64.26%) with 16 missing) in 15 countries. The sample sizes and demographics for each country are in Table 1 and represent a subgroup of a 28-country study (Schermer et al., 2019b). In the original 28-country study, samples were chosen with the goal of sampling as many countries as possible. Of the 28 country samples, the 15 countries with acceptable measurement properties for the humor scales, such that the coefficient alpha value was greater than .60 for each of the four scales, was retained for this investigation with the correlations with loneliness. Participants completed questionnaires either online or paper-and-pencil (see Table 1) after indicating informed consent.

**Table 1:** Demographic Statistics for the Samples Across the 15 Countries

Country	Language	Data collection procedure	*N* females	*N* males	Age - *M* years (*SD*)	Age range
Brazil	Portuguese	Online	209	95	28.76 (11.38)	18–71
Bulgaria	Bulgarian	Paper-pencil	128	131	19.94 (1.49)	17–33
Canada	English	Online	109	119	24.30 (5.20)	18–55
Chile	Spanish	Online	164	69	20.97 (3.10)	17–49
Colombia	Spanish	Online	142	114	21.06 (3.22)	18–48
Estonia	Estonian	Paper-pencil	153	215	24.28 (6.96)	18–49
Germany	German	Online	258	75	26.83 (6.56)	17–57
Poland	Polish	Online	167	78	23.75 (4.43)	18–40
Portugal	Portuguese	Online	375	94	22.82 (7.46)	17–78
Russia	Russian	Online	189	125	19.64 (1.64)	18–29
Serbia	Serbian	Online	302	102	21.73 (4.86)	18–52
South Korea	Korean	Paper-pencil	96	88	21.77 (2.13)	18–27
Spain	Spanish	Online	226	100	23.71 (5.84)	18–55
Ukraine	Ukrainian	Online	270	71	26.93 (9.82)	17–82
United States	English	Online	233	188	26.75 (3.26)	19–55

### Materials

The Humor Styles Questionnaire (HSQ; Martin et al., 2003) assesses four humor styles: affiliative, aggressive, self-enhancing, and self-defeating. The HSQ comprises 32 items (8 items for each of the four humor styles), responded to using a 1 (*definitely disagree*) to 7 (*definitely agree*) Likert-type scale. The HSQ has shown good internal consistency in previous research (alphas for subscales ranging from .77 to .81; Martin et al., 2003). The internal consistencies of the four HSQ scales for each of the country samples are in Table 2 and, as stated above, are each above .60.

**Table 2:** Scale Descriptives for the Four Humor Style Scales and Loneliness for Each Country

Country	Affiliative *M* (*SD*, α)	Aggressive *M* (*SD*, α)	Self-enhancing *M* (*SD*, α)	Self-defeating *M* (*SD*, α)	Lonely *M* (*SD*, α)
Brazil	44.47 (8.58, .84)	24.17 (7.36, .63)	34.11 (9.90, .82)	26.75 (9.69, .80)	5.59 (1.88, .82)
Bulgaria	44.48 (7.52, .73)	27.43 (7.97, .62)	35.32 (8.68, .71)	30.02 (7.89, .64)	4.76 (1.60, .76)
Canada	45.82 (6.74, .80)	28.78 (8.02, .73)	33.92 (9.52, .84)	28.12 (8.62, .78)	5.32 (1.76, .81)
Chile	42.76 (8.31, .82)	25.71 (7.94, .71)	35.78 (8.90, .81)	27.70 (8.46, .75)	5.71 (1.94, .81)
Colombia	41.06 (8.30, .78)	24.01 (7.51, .67)	35.77 (9.22, .81)	23.94 (8.21, .77)	5.15 (1.90, .82)
Estonia	44.26 (7.13, .80)	29.08 (7.56, .71)	35.48 (8.13, .78)	28.04 (7.76, .75)	4.86 (1.63, .76)
Germany	43.43 (8.23, .85)	26.71 (7.22, .66)	34.05 (9.16, .83)	25.54 (9.56, .85)	5.24 (1.49, .62)
Poland	42.86 (8.52, .85)	27.44 (7.79, .72)	34.10 (8.44, .80)	29.42 (8.72, .79)	5.67 (1.90, .82)
Portugal	43.57 (7.68, .81)	24.32 (6.73, .61)	33.33 (9.65, .83)	23.66 (9.46, .83)	5.26 (1.80, .79)
Russia	41.88 (8.40, .80)	29.73 (7.57, .63)	34.05 (8.49, .74)	28.25 (8.03, .70)	5.19 (1.78, .80)
Serbia	46.06 (6.59, .75)	24.57 (7.42, .64)	36.85 (8.86, .76)	27.60 (9.17, .79)	5.53 (1.52, .62)
South Korea	40.95 (6.79, .82)	25.31 (6.57, .70)	32.09 (6.54, .70)	28.29 (.32, .77)	4.47 (1.50, .73)
Spain	43.80 (7.14, .78)	22.26 (7.10, .66)	34.45 (8.64, .79)	26.66 (8.03, .74)	5.24 (1.86, .82)
Ukraine	42.66 (8.20, .81)	24.96 (6.93, .63)	35.03 (8.68, .79)	23.84 (8.12, .76)	4.93 (1.74, .78)
United States	41.35 (8.99, .86)	27.84 (7.68, .71)	35.64 (9.20, .85)	27.37 (9.05, .82)	5.52 (2.14, .88)

To measure loneliness, we used the Three-item Loneliness Scale (TILS; Hughes et al., 2004), which was designed for the needs of large survey studies. The TILS scale consists of three items derived from the Revised UCLA Loneliness Scale (Russell et al., 1980) which were first selected from a factor analysis and then adapted to the interview format (e.g., “How often do you feel left out?”). Items are administered as questions in the second person and participants provide answers using a 3-point scale (1 = *hardly ever*, 2 = *some of the time*, 3 = *often*). In the current study, German, Polish, and Spanish adaptations (Hawkley et al., 2016; Rico-Uribe et al., 2016) and newly prepared language versions of the TILS (Portuguese, Bulgarian, Estonian, Russian, Serbian, Korean, and Ukrainian) were used. The internal consistencies of the TILS are in Table 2 and Cronbach’s coefficient α was between .62 and .88 across countries.

### Statistical Analyses

After descriptive and correlative results, the relationship between humor and loneliness was tested using structural equation models. We examined how the four humor styles predict loneliness, while controlling for effects of gender and age. Therefore, loneliness was modelled as a latent variable with the three items of the TILS serving as ordinal indicators. The first loneliness item was used as a reference variable for identification purposes. The four humor style scores were computed from the HSQ and the sum scores were regressed on loneliness. Finally, gender and age were also regressed on loneliness. Through this regression, the relation between gender and age on loneliness was modelled and thereby controlled. All exogenous manifest variables were allowed to correlate.

In our model, we conceptualize loneliness as a latent variable and examine how the humor style scores, age, and gender predict loneliness. It should be noted that the same model may also be interpreted as a MIMIC-model (for an overview cf. Bollen & Bauldry, 2011; Strube, 2015). In a MIMIC-model, the latent variable would represent a causal indicators model of the four humor styles. This would be consistent with the literature on humor styles, which does not assume a second-order factor of humor (Martin et al., 2003). The three loneliness items are interpreted as three outcome variables. These are two competing views of the same latent variable. To harmonize these competing views, we 1) considered the latent variable as latent loneliness for ease of interpretation, but 2) interpreted the factor loadings of the three loneliness items as three regression weights for three indicators as outcomes.

As the data was collected across 15 countries, there is a hierarchical data structure that should be considered. In a first step, we inspected the intraclass correlation (ICC) of all manifest variables in the model across the countries. This ICC can be interpreted as how much percentage of the variance within a variable can be explained solely by the country affiliation. If this percentage is substantial (e.g. Hox et al. (2010) suggest 5% to be low and 15% to be high), then the hierarchical structure of the data needs to be considered. The ICCs are given in Table 3. As some humor styles varied substantially across countries (highest ICC for *aggressive* = 7.2%), we concluded that the hierarchical data structure needed to be considered for the humor styles. In addition, the covariates gender and age had sizeable ICCs reflecting that the samples were not entirely comparable across countries in age and gender. This difference is a limitation of our study and not a statistical artefact calling for correction. As the items assessing loneliness had negligible ICCs (ICCs ≤ .030), we concluded that the hierarchical data structure did not need to be considered for loneliness.

As 15 countries are not a sufficient number of clusters to warrant multi-level structural equation modelling, we estimated all models two-fold: 1) without considering the hierarchical data structure; 2) accounting for the hierarchical data structure by group mean centering the humor styles. Group mean centering removes the country level variance from the data (Enders & Tofighi, 2007). We thereby account for mean differences in humor styles across countries. Similar results across models with and without group mean centered humor styles would point to effects not being driven by country-level differences; non-similar results would suggest the opposite.

All models were estimated in R (R Core Team, 2018) with the *lavaan* package (Rosseel, 2012). The loneliness items have three response categories. Consequently, the loneliness items were treated as ordinal indicators. The models were therefore estimated with WLSMV (Lei, 2009; Savalei & Rhemtulla, 2013) with the loneliness items as ordered variables. Gender was dummy coded (0 = female, 1 = male) and considered as an ordinal variable. Model fit was assumed with a non-significant chi-squared statistic (Greiff & Heene, 2017), CFI ≥ .95, RMSEA < .06 and SRMR < .08 (Hu & Bentler, 1999; Marsh et al., 2004). Should model fit not be achieved, modification indices were inspected to point to the source of the misfit and to propose an alternate model. The critical ratio test was used to determine if parameters estimates of the models were significant (*p* < .05). The data and R-script are available at the Supplementary Materials section.

**Table 3:** Descriptive Statistics and Correlations Between the Humor Styles, Loneliness and the Covariates Gender and Age

		Descriptive Statistics								Humor Styles				Covariates		Loneliness		
Variable	Details	*n*	*M*	*SD*	ICC	Min	max	skew.	kurt.	Affiliative	Aggressive	Self-enhancing	Self-defeating	Gender	Age	Total	company	Left out
Humor Styles	Affiliative	4666	43.36	7.99	.033	8	56	-0.71	0.33
	Aggressive	4669	26.09	7.70	.072	8	55	0.18	-0.19	.12
	Self-enhancing	4656	34.75	8.96	.013	8	56	-0.07	-0.34	.43	.10
	Self-defeating	4671	26.81	8.85	.043	8	56	0.23	-0.29	.15	.30	.20
Covariates	Gender	4685	1.36	0.48	.060	0	1	0.61	-1.63	.02	.21	.08	.11
	Age	4701	23.75	6.65	.161	17	82	2.58	9.29	-.04^a^	-.09	.08	-.09	.02^a^
Loneliness	Total	4700	1.75	0.6	.031	1	3	0.48	-0.69	-.25	.01^a^	-.24	.20	-.06	-.08
	companionship	4699	1.86	0.70	.024	1	3	0.21	-0.96	-.17	.01^a^	-.17	.15	-.06	-.07	.80
	left out	4695	1.71	0.71	.030	1	3	0.48	-0.94	-.22	.02^a^	-.22	.18	-.06	-.07	.86	.54
	isolated	4698	1.68	0.73	.027	1	3	0.59	-0.95	-.25	.00^a^	-.22	.16	-.03^a^	-.05	.85	.50	.63

*Note*. Gender (0 = female, 1 = male). Loneliness: Total = total score of the TILS, companionship/company = *How often do you feel that you lack companionship?*; left out = *How often do you feel left out?*; isolated/iso. = *How often do you feel isolated from others?* For humor styles and loneliness, higher values mean higher standing on the trait. Possible range of the humor styles between 8 and 56, between 1 and 3 for loneliness.

Not significant (*p* ≥ .05).

## Results

### Descriptive Results

Table 3 shows the descriptive statistics and correlations between the humor styles, loneliness, and the covariates gender and age for the complete sample. As stated above, Table 2 gives the descriptive statistics and internal consistency estimates for the four humor styles and the loneliness scores separated for the countries. Table 4 presents the correlations between loneliness with the humor style scores, age, and gender, for each country sample.

**Table 4:** Correlations Between Loneliness With Humor Styles, Age, and Gender for Each Country

Country	Affiliative	Aggressive	Self-enhancing	Self-defeating	Age	Gender
Brazil	-.16*	.02	-.32*	.25*	-.28*	-.02
Bulgaria	-.26*	-.02	-.16	.07	-.12	-.01
Canada	-.36*	.04	-.14	.27*	.05	-.04
Chile	-.26*	.05	-.26*	.11	-.10	.02
Columbia	-.22*	-.03	-.17*	.17*	-.16	-.11
Estonia	-.31*	-.10	-.26*	.14*	-.11	.01
Germany	-.25*	-.02	-.21*	.20*	.07	.02
Poland	-.34*	.08	-.30*	.27*	-.16	-.09
Portugal	-.23*	.01	-.37*	.32*	-.18*	-.02
Russia	-.22*	.03	-.21*	.15*	-.01	-.15*
Serbia	-.29*	.06	-.21*	.20*	-.10	.10
South Korea	-.28*	.06	-.11	.21*	-.03	-.09
Spain	-.26*	.08	-.15*	.21*	-.04	-.04
Ukraine	-.28*	.01	-.33*	.14*	-.11	-.06
United States	-.32*	.03	-.36*	.23*	-.12	-.13*

*Note*. Gender: 1 = female, 2 = male.

*p < .01.

Across all of the country samples, the highest scores were for the affiliative humor style, followed by the self-enhancing humor style, with the lowest scores for the aggressive and self-defeating humor styles. For individual country comparisons, affiliative humor style scores varied significantly across the countries; *F*(15, 4936) = 11.14, *p* < .001. Serbia had the highest mean for affiliative humor style (significantly, following Bonferroni post-hoc with *p* < .001, higher than the values for Chile, Colombia, Germany, Hungary, Poland, Portugal, Russia, South Korea, Ukraine, and the United States) and South Korea had the lowest mean (significantly lower than the values for Brazil, Bulgaria, Canada, Estonia, and Serbia). Aggressive humor style scores varied significantly across the country samples; *F*(15, 4939) = 26.20, *p* < .001. Russia had the highest mean for aggressive humor style (significantly (*p* < .001) higher than the values for Brazil, Chile, Colombia, Germany, Hungary, Portugal, Serbia, South Korea, Spain, and the Ukraine) and Spain had the lowest mean (significantly (*p* < .001) lower than the values for Bulgaria, Canada, Chile, Estonia, Germany, Poland, Russia, South Korea, Ukraine, and the United States). Self-enhancing humor style scores significantly varied across country samples; *F*(15, 4926) = 5.34, *p* < .001. Serbia had the highest mean for self-enhancing humor style (significantly (*p* < .001) higher than the values for Portugal and South Korea) and South Korea had the lowest mean (significantly (*p* < .001) lower than the values for Hungary, Serbia, and the United States). Self-defeating humor style scores varied significantly across the country samples; *F*(15, 4941) = 15.45, *p* < .001. Bulgaria had the highest mean for self-defeating humor style (significantly (*p* < .001) higher than the values for Brazil, Colombia, Germany, Hungary, Portugal, Spain, and the Ukraine) and Portugal had the lowest mean (significantly (*p* < .001) lower than the values for Brazil, Bulgaria, Canada, Chile, Estonia, Poland, Russia, Serbia, South Korea, Spain, and the United States). Loneliness scores varied significantly across country samples; *F*(15, 4962) = 10.30, *p* < .001, with the lowest scores for the South Korean sample (significantly (*p* < .001) lower than the values for Brazil, Canada, Chile, Germany, Hungary, Poland, Portugal, Russia, Serbia, Spain, and the United States) and the highest scores for the sample from Chile (significantly (*p* < .001) higher than the values for Bulgaria, Estonia, South Korea, and the Ukraine).

Overall, there was only a small relationship between loneliness and gender, with men scoring slightly lower on the loneliness scale. As evident in the correlations in Table 4, this small gender difference was mostly driven by the samples from Russia and the United States, demonstrating a significant point-biserial correlation with women scoring higher on the loneliness scale. There was also a small relationship between loneliness and age, with older participants feeling less lonely, with the correlations reaching significance for the samples from Brazil and Portugal. When the possible curvilinear relationship between age and loneliness was assessed, it was found that the correlation between loneliness and the age value squared was not significantly different, and was slightly lower, than the linear correlations. As such, the linear correlations between age and loneliness are reported.

As reported in Table 3, lonelier participants used more self-defeating humor and less affiliative humor and had lower scores on the self-enhancing humor style scale. No significant relationship between loneliness and the aggressive humor style was found for the complete sample. When individual country samples were examined (see Table 4), consistently a negative correlation was between loneliness and the affiliative humor style. Although not always reaching statistical significance, there were negative correlations between the self-enhancing humor style and loneliness. Similarly, the self-defeating humor style had positive correlations with loneliness although for some of the samples, the values did not reach statistical significance. For each sample, a non-significant correlation was between loneliness and the aggressive humor style.

### Relationship Between Humor and Loneliness

**Figure 1:**
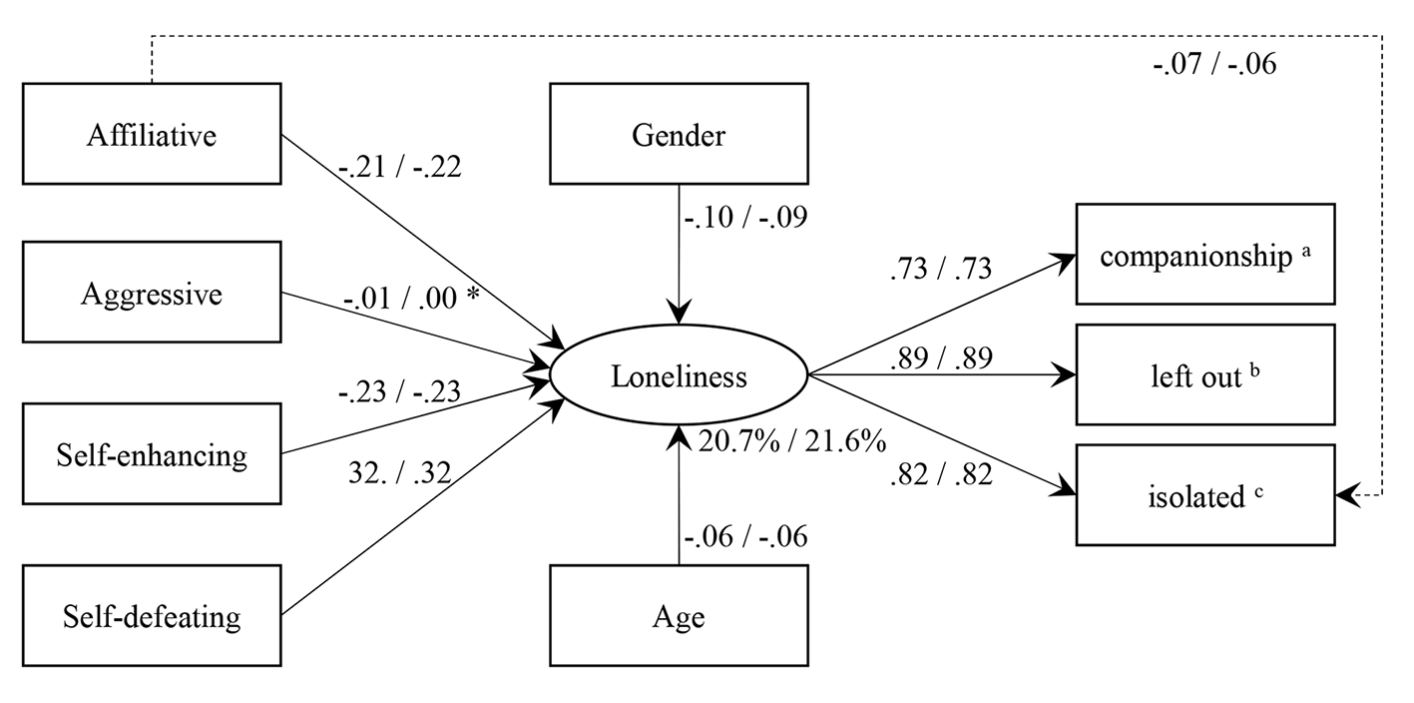
Structural Equation Model for Testing the Relationship Between the Humor Styles and Loneliness *Note*. Parameters on the left of the slash are derived from the uncentered model, parameters on the right from the centered model. The dashed path was added after the initial model showed misfit. All estimated are standardized. The parameter of gender was standardized only on the latent variable and can therefore be interpreted as Cohen’s *d*. *non-significant (p ≥ .05) ^a^How often do you feel that you lack companionship? ^b^How often do you feel left out? ^c^How often do you feel isolated from others?

The structural equation model in Figure 1 was fitted to assess the relationship between the humor styles and loneliness. First, this model was fitted with uncentered humor styles. The uncentered model did not fit the data, χ^2^(12) = 39.232, *p* < .001, RMSEA = .022, SRMR = .011, CFI = .998. Inspection of the modification indices revealed that the affiliative humor style had an influence on the feeling of being isolated from others over and above the latent variable. Adding this path to the model resulted in a good model fit, χ^2^(11) = 16.514, *p* = .123, RMSEA = .010, SRMR = .008, CFI = 1.00.

Second, the model was fitted with group mean centered humor styles. This model did also not fit the data, χ^2^(12) = 38.141, *p* < .001, RMSEA = .022, SRMR = .011, CFI = .998. Again, modification indices indicated that the misspecification was a missing effect of the affiliative humor style on the feeling of being isolated from others over and above the latent variable. Adding this path to the model made the model fit the data, χ^2^(11) = 15.466, *p* = .162, RMSEA = .009, SRMR = .008, CFI = 1.00.

In the last step, the model parameters shown in Figure 1 where inspected. Both affiliative and self-enhancing humor style were related to feeling less lonely. By contrast, the self-defeating humor style was related to feeling lonelier. An effect of the aggressive humor style could not be found. All but the aggressive humor styles strongly affected all three outcomes—lacking companionship, feeling left out, and feeling isolated from others. The strongest effect was given for the feeling of being left out. Furthermore, the feeling of being isolated from others was additionally reduced by a higher affiliative humor style. The covariates gender and age were both significant yet their effects negligible. These negligible effects suggest that being male and older was related to being slightly less lonely. The results from the centered and uncentered models were nearly identical. Therefore, we conclude that the relationship between humor styles and loneliness was not driven by country-level differences.

## Discussion

This study examined the relationships between humor styles and loneliness for men and women from samples in 15 countries. Consistently, the pattern suggests that individuals who use the affiliative humor style are less lonely. This pattern is as hypothesized, as the affiliative humor style is an adaptive style and reflects using humor to improve relationships with others (Lefcourt, 2001; Martin et al., 2003), and, as reviewed in the introduction, lonely individuals tend to report poor social bonds with others (Maes et al., 2016).

Less robust, but consistent, was the hypothesized negative relationship between loneliness and the self-enhancing humor style. As reviewed in the introduction, the self-enhancing humor style is intrapersonal and involves using humor to “cheer” one’s self, which alleviates negative states such as stress (Kuiper et al., 1993; Martin et al., 2003). One possibility for the less robust relationship between self-enhancing humor style and loneliness, when compared to the relationship between loneliness and the affiliative humor style, is the focus of the humor. Affiliative, by nature, is a humor style dealing with others. The self-enhancing humor style may alleviate feelings of stress but does not necessarily improve relationships with other people. Possibly applied researchers and clinicians may use these findings when providing guidance for lonely individuals and assist by training lonely individuals to focus on affiliative humor styles which are inclusive to the group.

As hypothesized, the self-defeating humor style positively correlated with loneliness (although for some of the individual country samples, the relationships did not reach statistical significance). These findings are consistent with Schermer et al. (2017), who suggested that because individuals who engage in self-defeating humor are making fun of themselves, this behavior is possibly a source of discomfort for others, which may, in turn, result in others avoiding someone who uses a self-defeating humor style. Future research may want to examine how others react to interacting with an individual who behaves with a self-defeating humor style.

Also consistent with past findings (Schermer et al., 2017), the aggressive humor style did not correlate significantly with loneliness. The aggressive humor style, as reviewed above, involves belittling others. If this belittlement is directed towards an out-group, and the individual engaging in the aggressive humor style perceives themself as being a part of an in-group, they may not feel particularly lonely as they are a part of a group. Schermer et al. (2019a) reported that individuals who utilize an aggressive humor style are more likely to have alcohol dependence problems. Possibly those with an aggressive humor style are unaware or uninterested in their feelings of being lonely or not and hence the non-significant correlations between the two constructs. Future research may wish to assess how individuals with aggressive humor styles feel about their relationships with others. If less interested in relationships with others, then those with the aggressive humor style may not even consider feelings of loneliness; a topic requiring future study.

The geographical coverage of the 15 countries was fairly broad in this study. Of interest was the rank order differences between the countries for the loneliness scores. In the present study, the sample from Chile had the highest loneliness scores and the sample from South Korea had the lowest loneliness scores. Why this difference occurred is hard to speculate about as South American countries have not been studied as extensively as other nations (Yang & Victor, 2011). Hofstede’s cultural categorization (Hofstede, 2001) describes both Chile and South Korea as collectivist societies, therefore the individualism versus collectivism findings with respect to loneliness (Barreto et al., 2021; Maes et al., 2016) would not apply. One possible influence could be the great differences in population density. According to Google.com, Chile has 26 people per square kilometer versus South Korea which has 527 people per square kilometer. Although, as stated in the Introduction, people can be in a crowd and still feel lonely, possibly being in a collectivist society and being close together helps to alleviate loneliness more than being in a sparsely populated country. Future research may want to further examine the possible factors which may cause similar countries on cultural levels to differ with respect to loneliness.

### Limitations

Despite the fact that the present study incorporates data from multiple countries, one of its limitations reports to major differences within the various samples. The study gathered responses from 15 different countries incorporating participants with different age ranges across the samples. For those samples with more restricted age ranges, possible robust correlations with loneliness and age may have been missed, especially for assessing non-linear correlations between age and loneliness. Furthermore, data were collected using different languages and different collection methods (paper and online). As stated in the Method section, not all of the translated scales had been independently verified and require further study. In addition, the HSQ was not designed to assess any one cultural or ethnic form of humor. Greater information will be gleamed in future studies which examine culture-specific uses of humor.

A second limitation to this study is the scale used to assess humor styles. The convergence and representativeness of the HSQ in terms of its conceptual foundations has been questioned by some researchers, leading to the need for further research to ensure the instrument’s validity (Heintz & Ruch, 2015). An additional limitation refers to the fact that the TILS scale does not switch between positive and negative wording, potentially leading to a ‘response pattern’—where participants might provide answers without taking the time to think carefully about each item before responding.

### Conclusions

Humor needs to be studied from the transdisciplinary perspective in order to take into account not only psychological determinants but also specific cultural and historical context of every culture. As the previous review suggests, the relations between humor styles and loneliness could be dependent on the specific cultural context. Of interest was the finding that the effects of country did not alter the fit of the model predicting loneliness, suggesting that for the 15 countries tested, the pattern of relations between humor styles and loneliness does appear to be “universal”.

## Supplementary Materials

The supplementary materials provided are the R code and dataset used in the research and can be accessed in the Index of Supplementary Materials below.



RogozaR.
KrammerG.
 (2021, August 19). Humor and loneliness across 15 countries.
[R script, dataset]. PsychOpen. https://osf.io/jhp6f


## Data Availability

Data is freely available at Supplementary Materials.
